# Choice of hydrogen uptake (Hup) status in legume-rhizobia symbioses

**DOI:** 10.1002/ece3.325

**Published:** 2012-08-09

**Authors:** Henry Annan, Amber-Leigh Golding, Yinping Zhao, Zhongmin Dong

**Affiliations:** 1 Department of Biology, Saint Mary's University923 Robie Street, Halifax, Nova Scotia, Canada, B3H 3C3; 2 Department of Biology, Xi'an University of Arts and Sciences168 TaiBai NanLu, Xi'an, Shaanxi Province, 710065, China

**Keywords:** Hup status, PGPR, rhizobium-legume symbiosis, root system morphology

## Abstract

The H_2_ is an obligate by-product of N-fixation. Recycling of H_2_ through uptake hydrogenase (Hup) inside the root nodules of leguminous plants is often considered an advantage for plants. However, many of the rhizobium-legume symbioses found in nature, especially those used in agriculture are shown to be Hup^−^, with the plants releasing H_2_ produced by nitrogenase activity from root nodules into the surrounding rhizosphere. Recent studies have suggested that, H_2_ induces plant-growth-promoting rhizobacteria, which may explain the widespread of Hup^−^ symbioses in spite of the low energy efficiency of such associations. Wild legumes grown in Nova Scotia, Canada, were surveyed to determine if any plant-growth characteristics could give an indication of Hup choice in leguminous plants. Out of the plants sampled, two legumes, *Securigera varia* and *Vicia cracca*, showed Hup^+^ associations. *Securigera varia* exhibited robust root structure as compared with the other plants surveyed. Data from the literature and the results from this study suggested that plants with established root systems are more likely to form the energy-efficient Hup^+^ symbiotic relationships with rhizobia. Conversely, Hup^−^ associations could be beneficial to leguminous plants due to H_2_-oxidizing plant-growth-promoting rhizobacteria that allow plants to compete successfully, early in the growing season. However, some nodules from *V. cracca* tested Hup^+^, while others were Hup^−^. This was similar to that observed in *Glycine max* and *Pisum sativum*, giving reason to believe that Hup choice might be affected by various internal and environmental factors.

## Introduction

Rhizobia species have been credited with the fixation of atmospheric nitrogen by forming symbiotic relationships with leguminous plants. This process occurs within nodules, which are formed by the invasion of these bacteria into plant tissues, and allows the plant to utilize compounded nitrogen for amino and nucleic acid formation. In return, the plant supplies the symbionts with energy-rich carbon from photosynthates (Liu et al. 2011). The reaction, catalyzed by the nitrogenase enzyme, is summarized as:


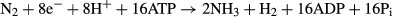


As shown, H_2_ is an obligate by-product of nitrogenase activity, representing about 5–6% of the plant's net photosynthesis (Dong and Layzell 2001). A breakdown of this calculation is shown in Table 1. In most cases, the H_2_ produced diffuses into the surrounding soil, signifying a loss of energy for the plant. For this reason, nitrogen fixation is often seen as energy inefficient.

**Table 1:** Energy and carbon cost of H_2_ production by nitrogenase

Item	Activity	Carbon distribution
1	Net photosynthesis	100%
2	Nodule CO_2_ evolution	15–25% of Item 1 (Layzell et al. 1979)
3	Nitrogenase activity	∼70% of Item 2 (Layzell et al. 1988)
4	H_2_ production	∼33.3% of Item 3 (Hunt and Layzell 1993)
		3.5–5.8% of Item 1

Studies have shown that some strains of rhizobia possess a H_2_ uptake (Hup) gene that codes for the enzyme hydrogenase. The hydrogenase enzyme allows the bacteria to recover some of the lost energy by oxidizing H_2_ (Dong and Layzell 2001; McLearn and Dong 2002). This results in an improved overall efficiency of the nitrogen-fixing process and increased nitrogen fixation. Soybeans, lupines, and cowpeas are among the most well-known legumes capable of forming symbiotic relationships with rhizobia that possess the Hup gene (Brito et al. 2005). Rhizobial strains or nodules that express uptake hydrogenase activity are termed Hup-positive (Hup^+^). Conversely, nodules that do not possess or express the Hup gene are termed Hup-negative (Hup^−^). Some reports have shown that the recycling of H_2_ significantly increases the nitrogen levels in legumes, consequently increasing the total yield (Dixon 1972; Schubert and Evans 1976; Albrecht et al. 1979).

In spite of the apparent benefits of H_2_ recycling, Hup^+^ symbioses are in the minority among surveyed legume microsymbionts. This seems to contradict evolutionary processes, which would be expected to favor the more efficient legumes. It is hence unclear why the natural and/or artificial selection of optimal N_2_-fixing bacteria have not reduced this energy loss in all legumes. The inconsistency suggests that releasing H_2_ into the soil may be beneficial to the plant. The positive effects that leguminous plants have on soil have been long exploited by farmers through crop rotations (Bullock 1992). More bountiful harvests are generally obtained when plants are allowed to grow on soil that had supported legumes in the previous growing season. It has been shown that the metabolism of H_2_ by H_2_-oxidizing bacteria can change the microbial community structure in soil (Zhang et al. 2009). This H_2_-treated soil has positive effects on plant growth as shown by Dong et al. (2003). Maimaiti et al. (2007) have also reported that these soil bacteria can promote root elongation and possibly increase nodulation.

The present study investigated the presence of uptake hydrogenase activity in wild legumes in the Canadian province of Nova Scotia. The plant root morphology was particularly observed as a possible indicator of Hup choice in leguminous plants.

## Methods

### Plant material collection

Several wild leguminous plants were randomly sampled from various locations within the Canadian province of Nova Scotia. Representative natural habitats for the wild legumes collected included coastal shores, roadsides, parks, and open fields. The sampling was performed during the summer months of late May to August when wild legumes typically start to flower (Roland and Smith 1969). This made for easier identification of legumes. Plants were carefully removed with soil attached to the roots in order to ensure that root nodules remained intact. Plants were transported to the laboratory in plastic bags and root nodules were immediately excised, washed, and tested for their HUP status. Where nodule excision could not be performed immediately after uprooting, the plants were kept aerated and in moist soil so that they remained alive until the nodules were excised.

### Methylene blue reduction assay

The method described by Lambert et al. (1985) and Zhang (2006) was used for the methylene blue reduction assay. All nodules were thoroughly washed in water to remove soil particles and excess plant matter. The number and distribution of root nodules varied from plant to plant, but were generally no less than ten per legume. Washed nodules were then placed in small labeled seed germination plates with filter paper that had been soaked in a methylene blue reduction solution of 200 mmol/L iodoacetic acid, 200 mmol/L malonic acid, 2.5 mmol/L MgCl_2_, 50 mmol/L K_2_HPO_4_, 10 mmol/L Methylene Blue dye, and adjusted to pH 5.6 with KOH. The nodules were distributed evenly on the plates, crushed and left to incubate in air for 15 min. The plates were then placed under a 100% H_2_ atmosphere in a sealed gas chamber overnight at room temperature. Plates were removed and photographed using a Canon PowerShot S21S digital camera. The presence of colorless zones around each nodule represented uptake hydrogenase activity (Hup^+^) while the absence of colorless zones represented Hup^−^ microsymbionts.

## Results

### Hup status of plants

**Figure 1:**
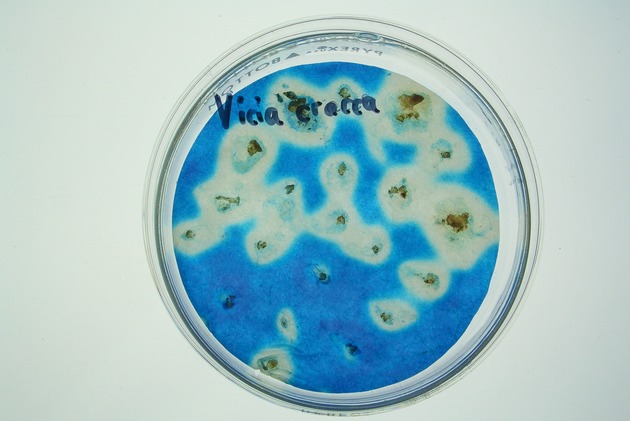
Hup^+^ root nodules of *Vicia cracca* as evidenced by presence of white bands post H_2_ incubation with methylene blue assay solution.

**Figure 2:**
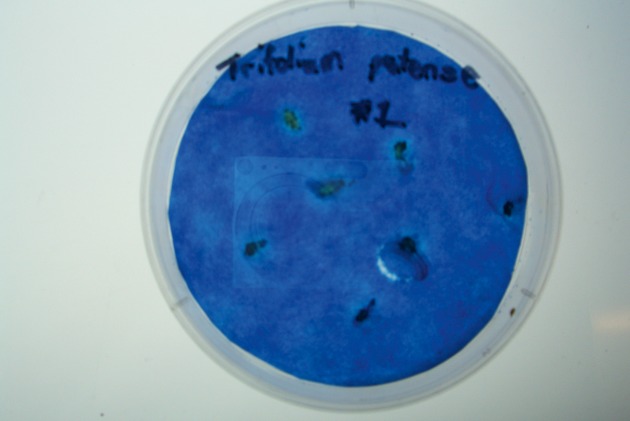
Hup^−^ root nodules of *Trifolium pratense* as evidenced by the absence of white bands post H_2_ incubation with methylene blue assay solution.

Eighteen species of wild leguminous plants belonging to eight different genera were investigated. The plants were taken from seven different municipalities in the Nova Scotia provincial area. Root nodules from soybean plants inoculated with the Hup^+^ (JH) and Hup^−^ (JH47) strains of *Bradyrhizobium japonicum* were used as positive and negative controls, respectively. The majority of the plants surveyed in this experiment had Hup^−^ nodules, while only two species possessed Hup^+^ nodules. These were *Vicia cracca* (the tufted vetch) and *Securigera varia* (the crown vetch). The reduction of the methylene blue by the Hup^+^ *V. cracca* root nodules as compared with the Hup^−^ nodules of *Trifolium pratense* is shown in Figures 1 and 2. The nodule Hup statuses detected in this survey are listed in Table 2,

**Table 2:** Hup statuses of surveyed wild legumes in Nova Scotia

Genus	Species [number species collected in location]	Common name	HUP status	Location collected
*Apios*	*A. americana* Medic.	Groundnut	(−)	Fancy Lake
*Lathyrus*	*L. maritimus* (L.) Bigelow	Beach pea	(−)	Little Tancook
	*L. maritimus* (L*.)* Bigelow	Beach pea	(−)	Lawrencetown
*Lotus*	*L. corniculatus* L.	Birdsfoot-trefoil	(−)	Halifax
	*L. pedunculatus* Cav.	Tick trefoil	(−)	Dundee
*Lupinus*	*L. polyphyllus* Lindl.	Garden lupine	(−)	Halifax
	*L. nootkatensis* Donn	Lupine	(−)	Halifax
*Medicago*	*M. sativa* L.	Alfalfa/ Lucerne	(−)	Truro
	*M. lupulina* L.	Black meddick	(−)	Truro
*Trifolium*	*T. arvense* L.	Rabbitfoot clover	(−)	Dundee
	*T. arvense* L.	Rabbitfoot clover	(−)	Halifax
	*T. aureum* Polich	Large hop trefoil	(−)	Halifax
	*T. campestre* Schreber	Low hop clover	(−)	Dundee
	*T. campestre* Schreber	Low hop clover	(−)	Halifax
	*T. hybridum* L.	Alsike clover	(−)	Dundee
	*T. pratense* L.	Red clover	(−)	Halifax
	*T. pratense* L.	Red clover	(−)	Marie Joseph
	*T. repens* L.	White clover	(−)	Halifax
	*T. repens* L.	White clover	(−)	Marie Joseph
*Vicia*	*V. cracca* L.	Tufted vetch	(−/+)	Halifax
	*V. cracca* L.	Tufted vetch	(−)	Newport
	*V. cracca* L.	Tufted vetch	(+)	Truro
	*V. sepium* L.	Hedge-vetch	(−)	Dartmouth
	*V. sepium* L.	Hedge-vetch	(−)	Marie Joseph
*Securigera*	*S. varia* (Formerly *Coronilla varia*)	Crown vetch	(+)	Halifax

### Physical characteristics of plant roots

The roots of the surveyed plants were generally characterized by thin lateral roots. *Trifolium* spp. in particular possessed especially delicate roots systems except for the perennial *T. hybridum* whose roots were noticeably larger as is expected of perennial root systems. Similar root sizes were observed in *Lupinus* spp. *Medicago sativa* as well as the *Vicia* and *Lotus* spp. had robust main roots along with the thinner lateral root systems. Notable exceptions were the roots of the beach pea, *Lathyrus martimus*, and the crown vetch, *S. varia*, which exhibited robust root morphologies with heavy, deep taproots, and thick lateral roots.

Rhizomatous growth was observed in the *V. cracca* plants sampled. The perennial groundnut, *Apios Americana*, possessed tubers although the roots themselves were similar in size to the majority of the surveyed plants. These tubers are indicative of annual subterraneous growth of the legume as necessary for tuberous propagation. This is in spite of the qualification of groundnut as a perennial.

## Discussion

The loss of H_2_ to the soil from legume root nodules inoculated with uptake hydrogenase-deficient rhizobia is traditionally viewed as a disadvantage of Hup^−^ symbioses relative to their Hup^+^ counterparts. This perspective is based on previous assumptions that H_2_ released into the soil is emitted into the atmosphere in a similar way as other gases such as CO_2_ and N_2_O. It was discovered, however, that the H_2_ never does leave the plant-soil system (Conrad and Seiler 1979) but is instead consumed within 1–4cm of the originating root nodules (LaFavre and Focht 1983). Subsequent studies have confirmed that the uptake mechanism of H_2_ in soil is bacterial in nature (McLearn and Dong 2002). Various species of H_2_-oxidizing bacteria, some of which have been successfully isolated in the laboratory (Maimaiti et al. 2007), have exhibited plant-growth-promoting properties potentially qualifying them as plant-growth-promoting rhizobacteria (PGPR). These PGPR may represent a significant benefit of H_2_ released from Hup^−^ nodules (Dong et al. 2003).

Some of the isolated PGPR have been shown to possess the enzyme 1-aminocyclopropane-1-carboxylate (ACC) deaminase and/or exhibit rhizobitoxine activity (Maimaiti et al. 2007). The ACC deaminase acts by cleaving ACC, a precursor to ethylene, to form α-ketobutyrate, thus disrupting ethylene synthesis (Hontzeas et al. 2006). Rhizobitoxine, a chemical inhibitor, functions to block the action of ACC synthase and effectively interrupts ethylene formation (Sugawara et al. 2006). Ethylene inhibition is said to promote nodulation in most legumes (Hunter 1993) and increase root elongation (Maimaiti et al. 2007). These enhanced root elongation properties could play a critical role in the establishment of root systems early in growing season. A larger root system and an increased number of nodules provide plants with better access to soil nutrients (Shah et al. 1998) as well as fixed nitrogen. These observations help to explain the benefits of crop rotations in agriculture and also illustrate that, while the benefits of hydrogen in the soil are experienced by neighboring plants, these benefits may also last beyond the current season and into the next growing season.

These studies suggest then, that there is a significant advantage for Hup^−^ associations especially in those plants that need to establish root systems early in the season due to the positive effects of H_2_ gas released into the rhizosphere. Plants that require a reestablishment of root systems from growing season to growing season would benefit greatly from H_2_-rich soil. By selecting for Hup^−^ microsymbionts, the plants would be able to compete with plants whose root systems have already been established from the previous season. Rhizomatous plants, which also require fresh root growth from their rhizomes annually, would benefit from the promotion of root elongation by PGPR. This is supported by the results of this experiment in which a majority of the legumes surveyed tested Hup^−^. The delicate root structures of the surveyed plants would not persist well over the winter and would thus need to be regrown each season.

In a cooler temperate climate, such as that observed in Nova Scotia, the positive effects of PGPR on plant and root growth are particularly important because the growing season is short compared with more tropical climates. Although energy from sunlight is limited under such conditions, the loss of energy caused by the evolution of H_2_ is far outweighed by the plant's effort to be competitive, especially among other non-leguminous plants. For this reason, it becomes beneficial for Nova Scotia's legumes to form symbiotic relationships with rhizobia that lack uptake hydrogenase activity. This would explain why a majority of the plants surveyed in this study tested negative for Hup activity. The benefits of H_2_ gas release in the soil for such plants may also explain why evolutionary processes have not selected for Hup^+^ associations, but instead, perhaps, selected against them as observed in the plants surveyed.

There is a growing body of evidence of Hup^+^ symbioses occurring in leguminous and some non-leguminous but nodule-forming trees (Cai et al. 1993; Zou et al. 1995; Zou et al. 1998; Huang et al. 2004). Sellstedt and Lindbald (1990) reported H_2_-uptake activity in *Frankia* species associated with the speckled alder, *Alnus incana*. Similar *Frankia* associations have been observed in the tropical *Casuarina* spp (Sellstedt and Winship 1987). The leguminous black locust trees, *Robinia pseudoacacia*, have demonstrated Hup^+^ associations with rhizobium spp (Röhm et al. 1993). Nodules from certain strains of the South-American Roman Cassie, *Acacia caven*, have also been reported to actively recycle H_2_ (Frioni et al. 1998). The quick-root establishment in these trees at the beginning of the growing season is not of great importance as they have well-established root systems, which persist over the seasons. Therefore, enriching PGPR by releasing H_2_ would be undermined by the overall efficiency of energetic processes such as nitrogen fixation.

The Hup^+^ result obtained for *S. varia* in the current study suggests the occurrence of more established root systems for this plant over winter. Although not comparable in size to the roots of tropical and subtropical leguminous trees, the roots of *S. varia* were observed to be much more robust compared with many of the other plants sampled. This may be an indication of Hup^+^ associations in more robust root structures. While the beach pea, *Lathyrus maritimus* possessed roots, similar in size to *S. varia*, but still tested Hup^−^, it is important to note that the beach pea traditionally grows in the sandy terrain of coastal shores where soil nutrients are comparatively scarce and is therefore likely to benefit significantly from the presence PGPR.

One particularly interesting result was the Hup status of *V. cracca*, which tested Hup^+^ in some plants sampled and Hup^−^ in others. This has been observed in certain leguminous plants, such as the soybean and lupine. Up to 75% of commercially grown soybean, *Glycine max*, in the United States are associated with *Bradyrhizobium japonicum* strains that exhibit the Hup^−^ phenotype, while the remaining 25% are Hup^+^ (Uratsu et al. 1982). It has also been suggested that pea plants, *Pisum sativum*, may possess root and shoot factors that control the expression of hydrogenase uptake activity in their root nodules (Bedmar and Phillips 1984). Evidently, while some rhizobial strains are deficient in hydrogenase genes, others may not express the genes at all although they do possess them. In *Lotus pedunculatus* for example, where rhizome growth is apparently dependent on seasonal and environmental factors (Blumenthal and Harris 1998), the expression of the Hup gene may also depend on similar factors. This conclusion can also be applied to *S. varia*, although further sampling is required to determine if there are times when it also exhibits the other Hup phenotype.

The results of this study suggest that there are significant benefits of both Hup^+^ and Hup^−^ associations in plant roots. Hup choice in leguminous plants seems then to be, at least to some extent, dependent on plant morphology and growth patterns and environmental conditions. The presence of PGPR in H_2_-exposed soil is particularly advantageous for plants that require the quick establishment of root systems. This is especially the case for leguminous plants in the province of Nova Scotia where the growing season is short compared with other more tropical climates. Evolutionary pressures are therefore more likely to select for the Hup^−^ phenotype in order for the legumes to be successful during vegetative competition with other plants. This phenomenon is in contrast to other more subtropical legumes whose growth is not as limited by the climate. In plants with more robust root systems, where fast root growth in spring may not be as important, the Hup^+^ phenotype is more likely to be selected for. *Secugeria varia* tested Hup^+^ and its root structures were noticeably more robust than the other plants tested. The ability of *V. cracca* to show both Hup^+^ and Hup^−^ associations will require future experimental surveys on leguminous plants to determine to what extent some legumes actively inhibit the expression of the Hup gene in Hup^+^ microsymbionts. Furthermore, more extensive studies into the Hup statuses of wild legumes in tropical and temperate climates may also be performed in the future, and their results contrasted, to help advance the general understanding of Hup choice in wild leguminous plants.
